# Complete mitochondrial genome of the abalone shell-boring *Polydora hoplura* (Polychaeta, Spionidae)

**DOI:** 10.1080/23802359.2022.2047116

**Published:** 2022-03-02

**Authors:** Soon Jeong Lee, Sang-Rae Lee

**Affiliations:** a Aquatic Disease Control Division, National Fishery Products Quality Management Service (NFQS), Busan, Korea; b Marine Research Institute, Pusan National University, Busan, Korea

**Keywords:** Polychaeta, abalone shell-boring species, mitogenome, *Polydora hoplura*

## Abstract

*Polydora hoplura* Claparede 1868 (Polychaeta, Spionidae) is a mollusk shell-boring species which has been reported as a harmful species. In the present study, we sequenced and reported the mitochondrial genome (mitogenome) of *P. hoplura* from Korea. Its mitogenome was determined by long range PCR and primer walking. The mitogenome was found to be 17,707 bp in length and contained two ribosomal RNAs (rRNAs), 23 transfer RNAs (tRNAs), and 12 protein-coding genes which showed different gene arrangement with that in related mitogenomes of species of Sedentaria (Annelida). Interestingly, the *atp*8 gene was not found in the mitogenome of *P. hoplura*, whereas it is present in the mitogenomes of related species *P. brevipalpa* and *P. websteri*. Moreover, *nad*2 (3’ end) and *cox*1 (5’ end) genes overlapped in 23 bases. The mitogenome structure and gene contents of *P. hoplura* provide useful information on the evolution and phylogenetic relationship among polychaete species.

Species belonging to the genus *Polydora* (Spionidae, Polychaeta) have been reported as harmful mollusk shell-boring species (Radashevsky and Migotto 2017; Sato-Okoshi et al. 2017). In abalone aquaculture in Korea, Japan, and China, production has been seriously affected by shell-boring *Polydora* species (Radashevsky and Migotto 2017; Sato-Okoshi et al. 2013; Won et al. 2013). Therefore, accurate identification of spionid polychaetes using genetics is essential for monitoring shell-boring *Polydora* species. In our previous study, we identified two Korean *Polydora* species (*P. haswelli* and *P. hoplura*) by molecular phylogenetic analyses using a taxon-specific molecular marker (Lee et al. 2020).

A sample of *P. hoplura* analyzed in our previous molecular phylogenetic study was used for complete mitochondrial genome (mitogenome) sequencing (Lee et al. 2020; the sample was obtained from an aquaculture farm in Wando, Korea, in December 2015; 34°18′14.9″N, 126°46′14.7″E). The voucher specimen and the extracted total genomic DNA have been deposited in the Mokpo Regional Office, National Fishery Products Quality Management Service (NFQS) [the voucher number (NFQS-Polydora-2015-N001), contact person (Soon Jeong Lee, leesj73@korea.kr)]. Total genomic DNA was extracted from the body tissues of *Polydora* isolate from an abalone using DNeasy Blood & Tissue Kit (Qiagen, Germany). PCR products covering the complete mitogenome of *P. hoplura* were acquired using long range PCR. The complete mitogenome sequence was determined by primer walking for PCR products (Macrogen, Seoul, Korea). All Sanger sequencing data were finally assembled using Sequencer 5.4.6 (Gen Code, Ann Arbor, MI, USA) and Geneious Prime (https://www.geneious.com). Gene structure mapping and gene annotation were conducted using the BLAST search and ORF finder in GenBank (NCBI). The tRNAscan-SE 1.21 (Schattner et al. 2005) was also used to identify the tRNA gene regions. Reference sequences of related Annelida species were downloaded from GenBank, and the phylogenetic analysis was conducted using MEGA 7 (Kumar et al. 2016).

In the present study, the complete mitogenome of *P. hoplura* was determined with 17,707 bp in length (GenBank accession number MZ584801). The genome sequence data that support the findings of this study are openly available in GenBank of NCBI at [https://www.ncbi.nlm.nih.gov]. Its nucleotide composition was 29.1% A, 23.4% C, 14.3% G, and 33.2% T, and it showed a biased A + T ratio (62.2%). It consisted of two rRNA genes, 23 tRNA genes, 12 protein-coding genes, and one control region, and its architecture was similar to that of *P. brevipalpa* and *P. websteri* (Ye et al. 2021). Moreover, a second methionine tRNA (tRNA-Met) was also found in *P. hoplura* mitogenome (Zhong et al. 2008). Ye et al. (2021) detected intergenic noncoding regions (NCR) in *P. hoplura* mitogenome. Interestingly, the *atp*8 gene was not found in the mitogenome of *P. hoplura*, even though it was found in the mitogenomes of *P. brevipalpa* and *P. websteri*. Seixas et al. (2017) also reported the missing of *atp*8 gene in the mitogenome of *Spirobranchus giganteus* (Annelida: Serpulidae) (Figure 1). Therefore, the further study is need to examine the existence and its function of *atp*8 gene among mitogenomes of polychaete species. Moreover, the *nad*2 (3′ end) and *cox*1 (5′ end) genes were overlapped in 23 bases (14 bases in *P. brevipalpa* and *P. websteri*).

**Figure 1.**
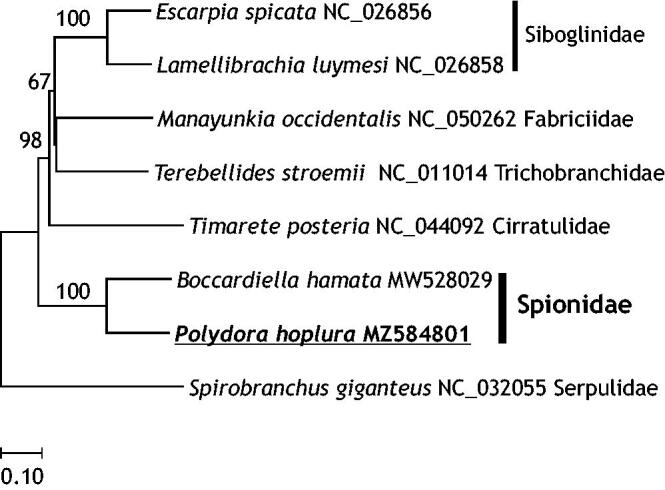
Molecular phylogenetic relationships among published mitogenomes of related Polychaeta species. In the neighbor-joining tree, genetic distances were calculated by Kimura-2 parameter method based on concatenated nucleotide sequences of protein coding genes (PCGs). Numbers above the branches represent bootstrap values (2000 replicates).

In summary, in the present study, we reported the complete mitogenome of *P. hoplura* and compared its interesting mitogenome features to those of other *Polydora* species. Our findings provide useful information for studying the evolution and phylogenetic relationships among polychaete species.

## Data Availability

The complete mitochondrial genome sequence of *Polydora hoplura* have been deposited in GenBank and openly available in GenBank of NCBI at https://www.ncbi.nlm.nih.gov/nuccore/MZ584801 under the accession no. MZ584801. The associated BioProject, SRA, and Bio-Sample numbers are PRJNA785067, SRR17081562, and SAMN23526757 respectively.
